# Inorganic Polysulfides and Related Reactive Sulfur–Selenium Species from the Perspective of Chemistry

**DOI:** 10.3390/molecules24071359

**Published:** 2019-04-06

**Authors:** Ammar Kharma, Marian Grman, Anton Misak, Enrique Domínguez-Álvarez, Muhammad Jawad Nasim, Karol Ondrias, Miroslav Chovanec, Claus Jacob

**Affiliations:** 1 Division of Bioorganic Chemistry, School of Pharmacy, University of Saarland, D-66123 Saarbruecken, Germany; s8amkhar@stud.uni-saarland.de (A.K.); jawad.nasim@uni-saarland.de (M.J.N.); 2 Institute of Clinical and Translational Research, Biomedical Research Centre, University Science Park for Biomedicine, Slovak Academy of Sciences, 845 05 Bratislava, Slovak; marian.grman@savba.sk (M.G.); anton.misak@savba.sk (A.M.); karol.ondrias@savba.sk (K.O.); 3 Instituto de Química Orgánica General, Consejo Superior de Investigaciones Científicas (IQOG-CSIC), 28006 Madrid, Spain; e.dominguez-alvarez@iqog.csic.es; 4 Cancer Research Institute, Biomedical Research Centre, University Science Park for Biomedicine, Slovak Academy of Sciences, 845 05 Bratislava, Slovak; miroslav.chovanec@savba.sk

**Keywords:** hydrogen sulfide, selenite, reactive sulfur species, reactive selenium species, polysulfides

## Abstract

Polysulfides (H_2_S_x_) represent a class of reactive sulfur species (RSS) which includes molecules such as H_2_S_2_, H_2_S_3_, H_2_S_4_, and H_2_S_5,_ and whose presence and impact in biological systems, when compared to other sulfur compounds, has only recently attracted the wider attention of researchers. Studies in this field have revealed a facet-rich chemistry and biological activity associated with such chemically simple, still unusual inorganic molecules. Despite their chemical simplicity, these inorganic species, as reductants and oxidants, metal binders, surfactant-like “cork screws” for membranes, components of perthiol signalling and reservoirs for inorganic hydrogen sulfide (H_2_S), are at the centre of complicated formation and transformation pathways which affect numerous cellular processes. Starting from their chemistry, the hidden presence and various roles of polysulfides in biology may become more apparent, despite their lack of clear analytical fingerprints and often murky biochemical footprints. Indeed, the biological chemistry of H_2_S_x_ follows many unexplored paths and today, the relationship between H_2_S and its oxidized H_2_S_x_ species needs to be clarified as a matter of “unmistaken identity”. Simultaneously, emerging species, such as HSSeSH and Se_n_S_8−n_, also need to be considered in earnest.

## 1. Introduction

Polysulfides (H_2_S_x_) represent an emerging class of reactive sulfur species (RSS) whose presence and numerous roles in biological systems have long been ignored. Only recently, more comprehensive studies in this field have uncovered some aspects of the facet-rich biological activity associated with such simple, still unusual inorganic molecules. Here, a certain focus has resided on the action of shorter chain polysulfides, such as H_2_S_3_ and H_2_S_4_. Notably, these species are not entirely new to biology—they have been around for a couple of decades, for instance as intermediates of natural garlic chemistry, although their production and impact in vitro and in vivo have been controversial [1]. Today, a couple of such shorter-chain polysulfides are accessible via chemical synthesis, and even though stability and hence purity is often an issue, their respective salts may be purchased from commercial suppliers and applied in biological studies [2].

With biological investigations of such species now underway, the considerable interest in the detection and activity of biological polysulfides in vivo has somewhat eclipsed their underlying chemistry. From the perspective of chemistry, the formation, physical and chemical properties, reactivity, and transformations associated with these species are far from trivial. Even the correct naming of S_x_^2−^ is not entirely uncontroversial within the biological community, with some colleagues referring to these molecules as “polysulfides” because of their inorganic nature and negative charges, whilst others prefer the term “polysulfanes”, pointing at the relevant IUPAC rules and also at certain natural metabolites, such as diallyltrisulfide (DATS) and diallyltetrasulfide (DATTS)—still risking a certain “charge confusion” with other “ides” on one side, and the non-charged organic “anes”, such as alkanes, on the other [3,4]. Ides and anes aside, and with due respect also to alternative naming practices, we will employ here the name “polysulfide” for inorganic species S_x_^2−^ and refer to “polysulfanes” in the context of organic RS_x_R’ or RS_x_H species (R ≠ H, x ≥ 2), being fully aware, of course, that this is not entirely uncontroversial.

Indeed, as part of this Special Issue on the emerging biological importance of polysulfides, we will take the readers on a brief and in part controversial detour to the basic and advanced chemistry of these species. Here, we will not simply “ab-use” chemistry in order to explain the processes underlying the biological occurrence and activity of polysulfides, such as S_2_^2−^, S_3_^2−^, S_4_^2−^, and S_5_^2−^. We will rather place chemistry first and explore the potential of the field of polysulfides from this perspective. We will then see which of this chemistry *de facto* translates into biology, and how. Projecting chemistry *a priori* onto biology will guarantee a broader, more speculative view of what may in fact be possible, and hence ensure that hitherto unnoticed species and reactivities may attract the attention of scientists, providing new possibilities for future discoveries.

## 2. Sources of Polysulfides

The initial question to be answered relates to if and how these inorganic polysulfides are formed in biology. Over the years, research into the catenation of sulfur has identified a wide spectrum of natural polymeric sulfur–sulfur species with sometimes impressive chain lengths reaching up to 30 and even more sulfur atoms [5]. Still, the presence of these long(er) chain species is mostly limited to oils and chemical products and may not apply to biology and medicine with their rather restricted conditions imposed by the physiological media, concentrations, timelines, temperatures and, notably, pH values.

**Figure 1:**
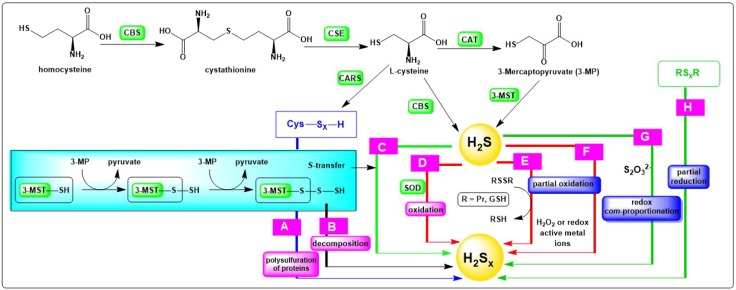
Chemical transformations potentially leading to the formation of inorganic polysulfides in vivo (R = glutathione or protein and x ≥ 2). Please see text for details.

From the perspective of chemistry, several pathways leading to H_2_S_x_ are imaginable, at least in theory, and some of the most prominent ones are summarized in Figure 1. It should be mentioned that this selection is focussed on reactions which are possible under physiological conditions. This does not mean that all of these reactions also take place in practice. It also does not rule out that other formation pathways may emerge in the future with the broadening of the information about the presence and role of such compounds in the biological systems.

The first and most accepted avenue leading to inorganic polysulfides begins with common sulfur-containing endogenous compounds, such as l-cysteine and 3-mercaptopyruvate, and enzymes associated typically with hydrogen sulfide (H_2_S) formation. Indeed, H_2_S these days is seen as “precursor” of biological polysulfides. Enzymatic formation, as illustrated in Figure 1, is therefore one central aspect of polysulfide formation [6]. H_2_S is rather abundant in biological systems and has been investigated for several decades as one of the crucial gaseous signalling molecules in biology [7]. It is produced endogenously mainly by four enzymes. The first enzyme is cystathionine β-synthase (CBS) which utilizes homocysteine as substrate, leading to the formation of cystathionine. The second enzyme, cystathionine γ-lyase (CSE or CGL), converts the aforesaid cystathione to l-cysteine, α-ketobutyrate and ammonia (NH_3_). Furthermore, l-cysteine is employed as substrate by CBS to release H_2_S with the simultaneous formation of pyruvate and NH_3_, or by cysteine aminotransferase (CAT) to form 3-mercaptopyruvate (3-MP). The fourth enzyme, 3-mercaptopyruvate sulfurtransferase (3-MST), employs 3-mercaptopyruvate as its substrate, thus leading to the formation of pyruvate and H_2_S. It has recently been discovered that cysteinyl-tRNA synthetases (CARS) employ cysteine as substrate to catalyse the production of cysteine persulfide (CysSSH) and polysulfides (CysS_x_SH), which represent other important precursors of H_2_S_x_. Cysteine persulfide subsequently interacts with thiols of proteins, causing polysulfuration of these proteins (Figure 1, Pathway A) [8]. Moreover, it has also been observed that 3-MST interacts with 3-mercapropyruvate and abstracts sulfur to constitute a persulfidated intermediate (3-MST-S_x_SH), which either degrades to form H_2_S_x_ directly (Figure 1, Pathway B) or transfers the sulfane–sulfur atom to other thiols or H_2_S to produce the corresponding persulfide species, including H_2_S_x_ (Figure 1, Pathway C) [8]. According to recent studies, it appears that H_2_S exerts many of its biological actions not directly, as traditionally assumed, and rather indirectly via the formation of H_2_S-derived polysulfides, such as hydropersulfides RSSH and higher order polysulfur compounds, i.e., RS_x_H, RS_x_R’, with R = glutathione or protein and x ≥ 3.

Besides such enzymatic transformations, the most obvious routes leading from H_2_S to H_2_S_x_ are via the —partial—oxidation of sulfide S^2−^. Such oxidations are feasible in chemistry, and the redox potential *E*^0^ = −0.447 mV vs. NHE for this transformation is also within the electrochemical potential range found inside the majority of biological systems [9,10,11]. There are also certain “footprints” detectable in the proteome which may be assigned as evidence to such direct sulfur oxidation processes, such as the presence of perthiol (RSS^−^) modifications in proteins, although these footprints are often not specific and could have also been caused by other agents and processes. In any case, the partial oxidation of H_2_S to H_2_S_x_, and not to elemental sulfur (S_8_) or sulfite (SO_3_^2−^), would require a fairly selective oxidizing agent or system, and it is still not entirely certain which specific oxidant(s) may accomplish this controlled redox transformation. Dioxygen and redox active metal ions, for instance, are probably an option (Figure 1, Pathway F). Superoxide dismutase (SOD) also catalyses the oxidation of H_2_S to produce H_2_S_2_, and, to a lesser extent, H_2_S_3_ and H_2_S_5_ (Figure 1, Pathway D) [9]. Notably, H_2_S_x_ species are also found as products of the reaction between H_2_S and nitric oxide ^•^NO, forging a chemical link between these two gaseous transmitters [12,13].

Furthermore, the partial oxidation of H_2_S may also proceed in the presence of milder oxidants, including disulfides, such as glutathione disulfide (GSSG) and certain protein-based disulfides (PrSSG, PrSSPr) (Figure 1, Pathway E). Indeed, the ability of H_2_S to reduce cellular disulfides directly, or in the presence of suitable oxidoreductase enzymes, is already established and is of paramount interest here, as it furnishes an avenue for widespread “inorganic” per- and polysulfuration of protein thiols. This pathway may be employed by cells to obtain persulfide modifications in and of proteins and enzymes (PrS_x_H), which subsequently may be central to cellular signalling [14,15].

We will return to the enormous biological potential of this kind of cellular redox signalling later on. Still, there is a caveat. Most cellular environments are reducing, and not oxidizing. Furthermore, recent, quite sophisticated studies employing protein-based intracellular redox sensors have noted that intracellular concentrations of GSSG and other disulfides are apparently considerably lower than estimated traditionally by more crude chemical staining techniques [16]. Whilst these measurements are still debated hotly, it appears that such widespread persulfide signalling cannot be the result of a simple chemical reaction scribbled down as “PrSSR + H_2_S → PrSSH + RSH”, possibly followed by “PrSSH + H_2_S → PrSH + H_2_S_2_”. These reactions, which embrace both reductive sulfuration of cysteine residues and the subsequent formation of inorganic polysulfides, may therefore be more prevalent and relevant in oxidizing compartments, such as the endoplasmic reticulum, where the concentrations of disulfides are considerably higher. Alternatively, PrSSH may be formed enzymatically, as mentioned already in the case of 3-MST above.

Considering that reduction, and not oxidation, dominates inside most mammalian cells, another avenue leading to the formation and/or presence of H_2_S_x_ in animals and humans becomes of interest, which involves the consumption of natural polysulfane products from fungi and plants, such as DATS and DATTS, found, for instance in garlic. Here, partial reduction, rather than oxidation, is required to convert the initial sulfur species into the relevant polysulfide (Figure 1, Pathway H). Intriguingly, plants tend to produce polysulfides via complex biosynthetic pathways, often involving other RSS, such as sulfenic acids and thiosulfinates, as precursors [17]. In garlic, for instance, the thiosulfinate allicin is produced first, which subsequently decomposes to yield, among others, some organic polysulfanes, which can be reduced by GSH to release inorganic H_2_S_x_ species [18]. It is therefore possible that some inorganic polysulfides appear in the human body as a result of the significant consumption of *Allium* vegetables rich in organic polysulfanes [19]. Still, this “formation pathway” is rather limited to particular and maybe even peculiar nutritional habits and diets, such as the Mediterranean diet, and, curiously, has also been caricaturized in certain movies [20]. Taken in reasonable moderation and with a pinch of salt, such a diet may be beneficial to human health [21]. A study in Italy and Switzerland spanning over two decades, for instance, has revealed that the recurrent consumption of *Allium* vegetables reduces the risk of developing cancer, especially in the digestive tract [22].

Still, let us return to chemistry. Here, one additional particularly promising pathway resulting in H_2_S_x_ formation involves “redox *com*-proportionation”, also referred to as “redox *syn*-proportionation”, i.e., converging oxidation states of the same element. In the case of sulfur, such a reaction involving sulfite (SO_3_^2−^) and H_2_S is employed industrially as part of the Claus process to produce elemental sulphur [4]. This reaction also provides an easy avenue to sulfur nanoparticles. In a more biological context, a similar reaction is found when HS^−^ reacts with inorganic thiosulfate S_2_O_3_^2−^ (Figure 1, Pathway G). This conversion results in the formation of longer sulfur–sulfur chains, and eventually in the cyclisation to S_8_, i.e., elemental sulfur. It is witnessed, for instance, in certain bacteria, such as ones of the genus *Thiobacillus*, which oxidize H_2_S in the presence of S_2_O_3_^2−^ and produce sulfur (nano-)deposits [23]. These bacteria are employed in the desulfuration of —industrial— waste waters [24]. They may also be interesting for the clean-up of “coal mine water”, i.e., waters rich in sulfur and collecting in abundant coal mines [25]. Once pumped up, these mine waters tend to pollute the surface water, such as rivers, and hence need to be dealt with as part of environmental sulfur chemistry.

Whilst such sulfur species are popular in the realm of microorganisms, S_2_O_3_^2−^ is not particularly common in mammalian biology, and despite the rather intriguing redox transformations it may initiate, is probably of minor importance [26,27,28]. The same also applies to SO_3_^2−^ and sulfate (SO_4_^2−^), both of which form a central part of the natural “sulfur cycle”, and hence also of potential H_2_S_x_ formation, still are difficult to reduce by mammals. If and how the human microbiome may be able to convert such oxidized species into polysulfides, and to which extent these charged molecules may then enter the human body and exert an(y) action there, is currently a matter of intense investigations [29,30]. As always, the gut microbiota may provide an attractive backdoor for unusual molecules, such as polysulfides, to enter the human body, before leaving it through the same [31].

**Figure 2:**
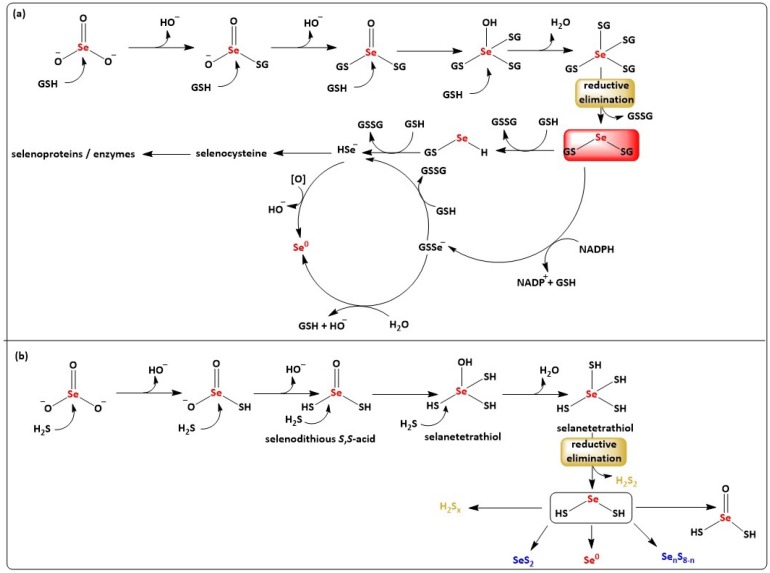
Suggested and in some parts still speculative pathways for the formation sulfur species and mixed sulfur-selenium species by sequential reduction of SeO_3_^2−^ in the presence of GSH (Panel **a**) and H_2_S (Panel **b**). Please see text for details.

Unlike SO_3_^2−^, whose presence is limited to certain food items, such as wine, dried fruits, and nuts, where it is added as preservative, and otherwise is of little relevance to human health, its selenium analogue (SeO_3_^2−^) is employed widely as a nutritional selenium supplement [32]. Selenite reacts readily with cellular reducing species, such as GSH, forming a wide range of intermediate reactive sulfur–selenium species, including selenodiglutathione (GSSeSG) (Figure 2, Panel (a)) [33]. This rather familiar, still often overlooked sequence of chemical transforms provides new avenues leading to inorganic polysulfides and mixed selenosulfides and *de facto* opens up an entirely new field of bioinorganic chemistry, in microorganisms, plants, mammals and humans. Indeed, the field of biologically relevant selenium molecules is limited to a handful of molecules, such as the ergothioneine analogue selenoneine found in blue tuna, and such inorganic selenosulfides may spice up this field of biological chalcogen research considerably [34].

## 3. Polysulfides and Mixed Selenosulfides Derived from H_2_S and SeO_3_^2−^

When H_2_S, in particular, is oxidized by SeO_3_^2−^, a wide range of sulfur and mixed-chalcogen species may be formed. It even appears that the reduced sulfide (sulfur in oxidation state−2) and the oxidized SeO_3_^2−^ (selenium in oxidation state +4) initiate an intricate—and rapid—interchalcogen redox cascade involving a range of highly reactive and often transient sulfur and selenium species.

Thanks to the “special relationship” which exists between sulfur and selenium, these transformations seem to be considerably faster when compared to the oxidation of H_2_S by O_2_ and in fact may generate chalcogen species responsible for the pro- and anti-oxidant activities hitherto assigned to either H_2_S or SeO_3_^2−^. Indeed, colleagues such as Kenneth R. Olson are already debating the tricky and sticky question if such “exotic” inorganic sulfur species may actually cause some or most of the biological actions traditionally ascribed to H_2_S as a matter of “mistaken identity” [35]. It is therefore of paramount importance to identify some of these less obvious RSS in biology and to establish their activity with respect to H_2_S.

When considering the interchalcogen redox reaction between H_2_S and SeO_3_^2−^ under the “mechanistic magnifying glass”, a series of subsequent nucleophilic substitution reactions of partially (de-)protonated H_2_S on SeO_3_^2−^ emerges, and necessarily so, as this reaction between selenium in the oxidation state +4 and sulfur in −2 clearly cannot proceed in just one step. Among others, this sequence of redox transformations results in the formation of species such as HSSe(O)SH, HSSeSH, Se_n_S_8−n_, HSSH, H_2_S_x_, and H_2_Se, some of which are familiar to sulfur biology already, whilst others are still more elusive (Figure 2, Panel (b)). Similar to many other small-molecule RSS, these species are also mostly anionic and hence nucleophilic under physiological conditions. Some of these species predicted *a priori* as part of this mechanistic map have been identified recently, for instance by Jung et al., who a couple of years ago reported that SeO_3_^2−^ in sulfide solution is reduced to elemental selenium or to selenium-sulfur precipitates (e.g., Se_n_S_8−n_), with sulfur-containing anions (e.g., S_2_O_3_^2−^, HSO_3_^−^, etc.) or elemental sulfur (S^0^), sulfide (S^2−^), and —notably—polysulfides (S_x_^2−^) as by-products, and with the exact identity and formula of these products depending on pH [36]. In contrast, other species of the mechanistic map, in particular the reactive tetra- and divalent selenium–sulfur intermediates, so far have remained more elusive [37,38,39].

Indeed, the literature on these important interchalcogen redox transformations is rather vague. In order to obtain more detailed information on these species, we have therefore ourselves seen the necessity to consider briefly the direct reactions of H_2_S with SeO_3_^2−^ and, for comparison, also with selenium tetrachloride (SeCl_4_). In agreement with the mechanistic map and also with previous studies, the UV-Vis spectra gradually change over time when the reduced sulfur compound, in this case Na_2_S, is added to an Na_2_SeO_3_ solution (or vice versa), indicating several ongoing chemical reactions between Na_2_S and Na_2_SeO_3_ which occur at different pH values [36].

Similar changes are observed when GSH is employed instead of Na_2_S. The redox nature of these interactions is obvious. There are no changes in the UV-Vis spectra when two oxidized (Na_2_SeO_3_, GSSG) or two reduced (Na_2_S, GSH) chalcogen species are added to each other, respectively.

**Figure 3:**
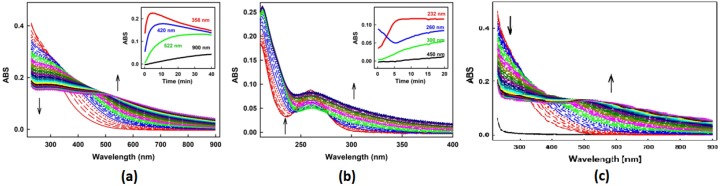
Representative time resolved UV-Vis spectra of the interaction of H_2_S or GSH with SeO_3_^2−^ or SeCl_4_. The interactions of: (**a**) H_2_S (100 µM) with SeO_3_^2−^ (100 µM), (**b**) GSH (100 µM) with SeO_3_^2−^ (100 µM), and (**c**) H_2_S (100 µM) with SeCl_4_ (100 µM). All samples were prepared and measured in 100 mM sodium phosphate, 100 µM DTPA buffer, pH 7.4, 37 °C. The spectra are represented in the form of solid and broken lines. The solid red line indicates the first spectrum after addition of H_2_S or GSH to SeO_3_^2−^ or. SeCl_4_, which is followed by gradual broken red, solid blue and gradually broken blue line which represent the spectra recorded every 30 s.

Interestingly, under the experimental conditions chosen, the interaction of H_2_S with SeO_3_^2−^ consists of two major, clearly distinguishable steps, whereby the changes in absorbance in the region below 520 nm e.g., at 358 nm and 420 nm, show a biphasic time-dependence, with a maximum after just 5–10 min, respectively, whilst the changes in the region between 520 nm and 900 nm gradually, and more slowly, increase over 40 min. It therefore appears that not just one major species is formed. Quite in contrast, there is clear evidence for the subsequent formation of two or more species, with one or more intermediate species formed rapidly, i.e., within ~5–10 min and stable for a few minutes, before these intermediate(s) absorbing at 420 nm react further to one or more species with broader absorbance at 520–900 nm. The more rapid reaction observed probably involves several nucleophilic substitutions on the selenium centre of SeO_3_^2−^, first resulting in the formation of two thioesters, followed by a tetravalent (HS)_4_Se intermediate which reacts to a more stable divalent selenium disulfane (HSSeSH). This selenium disulfane is captured by the spectra below 520 nm and can be deprotonated at neutral pH (Figure 3). Notably, inorganic polysulfide H_2_S_x_ species may also be released at this stage as part of the “reductive elimination” at the selenium redox centre.

The reactions and species shown in Figure 2, Panel (b) therefore agree with literature reports and various results obtained as part of our own investigations. Although inspired by the redox transformations of SeO_3_^2−^ with GSH shown in Figure 2, Panel (a), the transformations initiated by H_2_S are still considerably more complex, as sulfides have two “open valences” and hence may undergo more than one nucleophilic attack. The species formed, such as HSSeSH, are also prone to further reactions, including oxidation, reduction, decomposition, rearrangements and even thiol exchange.

Whilst some of the species postulated in Figure 2, Panel (b) are elusive, there is some direct evidence for the formation of the HSSeSH intermediate. First of all, the absorbance spectra obtained for the rapidly formed species in Figure 2, Panel (b) are similar to the ones obtained for anionic trisulfides (S_3_^2−^) and tetrasulfides (S_4_^2−^), pointing towards a similar, possibly inorganic selenotrisulfane intermediate, such as -SSeS-. Secondly, similar spectral changes were observed when tetravalent SeCl_4_ was employed, providing support for a tetravalent intermediate—whilst hexavalent SeO_4_^2−^, despite being more oxidized (selenium in oxidation state +6), did not interact with H_2_S under these experimental conditions. Thirdly, there are several significant analogies between this sequence of reactions and the ones observed for the already established interaction of GSH with SeO_3_^2−^ which follows a similar stoichiometry and timeline, forming a reasonably stable GSSeSG as intermediate, which indeed is analogous to HSSeSH [33,40,41]. Fourthly, the biological activities frequently observed for such mixtures (see below) point towards the presence of such reactive species, since sulfur and selenium nanoparticles, as solids, are only moderately reactive and biologically active, whilst H_2_S_x_ and similar mixed selenosulfides Se_n_S_8−n_^2−^ are sufficiently soluble and potent to exert a considerably biological activity [42,43,44]. And fifthly, the subsequent formation of elemental deposits may be rationalized by another “reductive elimination” in a species, such as HSSeSH, just as GSSeSG subsequently decomposes to elemental selenium and GSSG.

## 4. Nanoparticles

Indeed, as in the case of GSSeSG, the intermediate(s) formed by the reaction of H_2_S with SeO_3_^2−^ do not seem to be particularly stable in buffered, neutral aqueous solution for more than ~10 min. GSSeSG, for instance, is only stable at acidic pH, and “turns red” in buffered media due to the slow release of red elemental selenium and the formation of GSSG. This reaction, once more, constitutes a “reductive elimination” of an oxidized sulfur species at the selenium centre (Figure 2, Panel (a)). If applied to HSSeSH, such a reductive elimination would result in the formation of elemental selenium and, in this case, of H_2_S_2_. This “reductive elimination” to elemental, insoluble materials containing chalcogens, probably originating from HSSeSH, has been reported and has also been observed in our own investigations, with increases in absorbance between 520 and 900 nm indicative of light scattering, in this case caused by precipitation of fine particles of red elemental selenium or mixed elemental selenium–sulfur products [6,43,45,46].

Chemically and biologically, these particles are of particular interest as their respective compositions Se_n_S_8−n_ and structures resemble elemental forms of the chalcogens, i.e., S_8_ and Se_8_, and endow these particles with specific physical and chemical properties, redox reactivity and biological activity. Such nano- and microscopic, elemental formulations of these two chalcogens are often colloidal and seem to be employed widely in traditional agriculture as an eco-friendly fungicide to protect vineyards against *Botrytis cinerea* and also in medicine, for instance against the causes of dandruff [47,48,49,50]. Such elemental particles are also rather reactive on their own, as they may act as reductants and as oxidants, e.g., in the presence of thiols, forming disulfides and partially reduced Se_n_S_8−n_^2−^ compounds as part of the process.

## 5. Reactivity and Activity

Besides the formation and hence presence of polysulfides, reactivity with relevant biomolecules is essential for biological activity as the presence of polysulfides may go unnoticed otherwise. As mentioned already, considering the chemistry of such RSS *a priori* has some charm, as it provides a certain guidance to the ensuing biological activity. Indeed, from the perspective of chemistry, inorganic polysulfides are rather exceptional redox active molecules. As the sulfur atoms in these short and simple molecules are partially oxidized and possess different oxidation states, species of the formula H_2_S_x_ may act as reductants and oxidants, depending on the redox partners involved in each specific environment.

**Figure 4:**
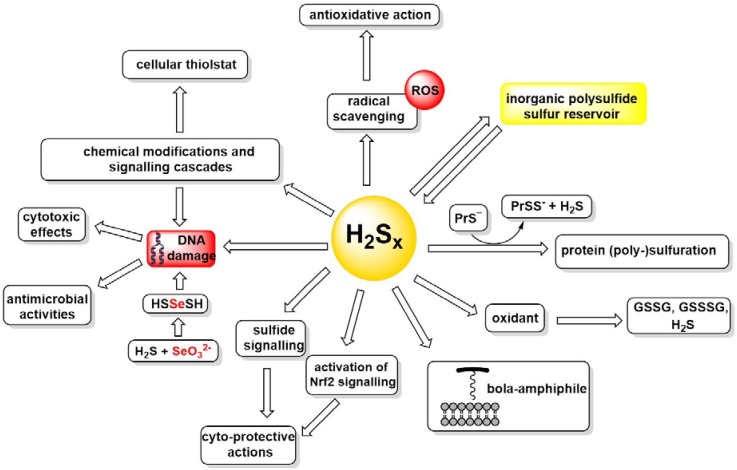
A necessarily incomplete and still speculative summary of the various chemical reactivities and biological activities associated with inorganic polysulfides. See text for details.

This kind of pronounced redox chemistry of polysulfides, often paired with metal binding and amphiphilic, surfactant-like properties typical for the longer sulfur–sulfur chains, endows these species with a pronounced biological activity [51]. At first sight, this activity often appears as a paradox, as polysulfides seem to act as protective and (cyto-)toxic species at the same time, still this conundrum is resolved once the redox amphoteric character described above is taken into consideration. A few examples of such biological actions established to date may therefore be considered as an appetizer to more extensive biological studies described elsewhere in this Special Issue (Figure 4).

On one side, S_x_^2−^ may reduce certain metal ions and also disulfides to form longer chain species, in the simplest case S_2x_^2−^, which may subsequently decompose to shorter chain polysulfides and, quite often, elemental sulfur S_8_. This kind of reductive behaviour is not dissimilar to the one of H_2_S, and here, H_2_S_x_ is considered increasingly as a clearly superior reducing agent [52,53]. Because of this pronounced reducing behaviour, some colleagues have recently considered H_2_S_x_ as the species *de facto* responsible for the reducing and metal binding activities traditionally assigned to H_2_S, which itself may simply be an imposter, a pre-cursor of such polysulfide chemistry, literally smelling here with other’s farts, as one tends to put it tellingly among our local mining community [35].

Na_2_S_4_exhibits a noteworthy —and direct— antioxidant activity. It is, for instance, a strong radical scavenging agent active against certain reactive oxygen species (ROS), the superoxide anion (O_2_^•−^) and the 2-(4-carboxyphenyl)-4,4,5,5-tetramethylimidazoline-1-oxyl-3-oxide (•cPTIO) radicals. Here, its activity is superior to the ones of H_2_S, glutathione and Trolox, i.e., the water-soluble analogue of vitamin E. Interestingly, Na_2_S_4_ demonstrates a bell-shaped potency to convert H_2_O_2_ into ^•^OH radicals and subsequently scavenges these ^•^OH radicals when applied at higher concentrations [43]. Similar results were also observed with the mixture of H_2_S and *S*-nitrosoglutathione, which produces several compounds, including polysulfides. A direct involvement of polysulfides in free radical scavenging pathways for modulating anti-oxidant/toxic biological activities may therefore be postulated on the basis of above-mentioned mechanisms [43].

Then again, the disulfide bonds in H_2_S_x_ may also serve as oxidants, for instance oxidizing GSH to GSSG and forming mixed disulfides such as GSSH, GSSSH, and possibly even H_2_S in the process. Interestingly, neither H_2_S nor SeO_3_^2−^ alone significantly cleaves plasmid DNA (pDNA). In contrast, a mixture of SeO_3_^2−^ and H_2_S turns highly damaging to DNA, increasing the cleavage of pDNA with the bell-shaped concentration ratio dependence similar to the one observed in the reduction of the ^∙^cPTIO radical. Once more, at the optimal stoichiometry of around 4:1 for H_2_S and SeO_3_^2−^, respectively, one or more highly reactive species, most likely including HSSeSH, are formed, which are able to attack DNA, probably directly and via a radical mechanism. The resulting oxidizing behaviour is reflected in a pronounced (cyto-)toxic activity associated with certain polysulfides. Indeed, prominent examples of such biological activities include antimicrobial activities against Gram-positive bacteria *Staphylococcus aureus* and Gram-negative bacteria *Escherichia coli*, *Pseudomonas aeruginosa*, and *Salmonella enteritidis* [51].

This pronounced toxicity confirms that polysulfides are not just “antioxidants”. In fact, the dominant behaviour in each case depends largely on the reaction partners present, and it is feasible that one particular polysulfide, such as S_4_^2−^, may act as reductant and oxidant within the same cellular environment. Notably, whilst such redox amphoteric molecules are not really rare in biology, it is certainly unusual that both, reduction and oxidation, proceed readily under similar physiological conditions.

Oxidation and reduction of H_2_S_x_, often in cahoots with H_2_S itself, has also led to another, rather interesting hypothesis of an “inorganic polysulfide sulfur reservoir” which serves as a source and sink of H_2_S, and obviously requires its very own sulfur redox chemistry [54]. If such a reservoir exists, and how extensive and physiologically relevant it may be, needs to be demonstrated.

Indeed, in mammalian systems, the “biological chemistry” of H_2_S_x_ is rather complicated, as it involves direct and also indirect reactions, chemical modifications and entire signalling cascades [55,56]. For instance, polysulfides activate the TRPA1 channel, regulate intracellular levels of calcium ions (Ca^2+^) during inflammation, influence PTEN/Akt/CREB signalling and mediate the cytotoxicity of 1,4-naphthoquinone by formation of the respective sulfur adducts [13,57,58]. Certain natural tetrasulfanes are also associated with the anti-proliferative effects in human breast cancer cells and demonstrate activity in the case of infections caused by bacteria resistant to antibiotics [51,59].

Notably, H_2_S_x_ also mediates the activation of Nrf2 signalling and protects Neuro2A cells against oxidative damage from lipid phosphatase, tensin homolog (PTEN) and glyceraldehyde 3-phosphate dehydrogenase (GAPDH). Similarly, cysteine persulfides and polysulfides generated by Na_2_S also protect SH-SY5Y cells from methylglyoxal cytotoxicity [42,60,61,62]. Here, the notion of persulfide formation and wider polysulfide/persulfide redox signalling emerges as a major intracellular signalling pathway in mammalian cells. This kind of “sulfide signalling” has considerable attraction, in chemistry and in biochemistry. It is probable that inorganic S^2−^, just as inorganic PO_4_^3−^, is able to modulate numerous biological processes, in this case primarily by modifying -SH groups, and mostly by *S*-persulfidation interactions with cysteine residues of proteins and enzymes of the “cellular thiolstat” to form protein persulfides (PrSSH) [63]. These and similar biological activities will be discussed by colleagues elsewhere in this Special Issue.

One should also mention that the -SeH group in selenocysteine represents another possible target, albeit the literature on this kind of chemistry is extraordinarily limited. A few compounds have been found to be effective precursors for persulfides (RSSH) and selenylsulfides (RSeSH) upon reacting with nucleophilic species. A cyclic acyl selenylsulfide, such as 3H-benzo[*c*][1,2]thiaselenol-3-one, for instance, reacts as an unique precursor for selenylsulfide (RSeSH), a selenol-based counterpart of persulfides [64]. Such selenylsulfides are expected to be important intermediates in sulfur redox processes in vivo involving selenocysteine or selenocysteine-containing proteins, still their chemistry and properties are largely elusive [65].

Besides serving as a “reservoir” for H_2_S, and a pivotal partner in polysulfide/persulfide signalling, inorganic H_2_S_x_ species have another couple of aces up their sleeves. They are, for instance, bola-amphiphilic molecules, i.e., structures with two polar head groups and a hydrophobic linker in between. These molecules, with their unique “cork-screw” structure can interact with and even insert into cellular membranes [66,67]. If such interactions are relevant in vivo still needs to be shown. Binding of and to metal ions, in small complexes and proteins, represents another possible mode of action which may need to be investigated and confirmed, as it may result in a wider metal-centred transport and modulatory activities (Figure 4).

In most of these cases, the relevant chemistry is feasible, still the detection of such H_2_S_x_ species in cells and cellular compartments, either directly or via their footprints, is difficult. Indeed, in order to understand the biological impact of H_2_S_x_, sensitive methods of time-dependent H_2_S_x_ detection in situ and in cells are required and several such approaches have been described in recent reviews [68,69,70]. A sensitive LC-MS/MS-based method, for instance, has been reported which employs a commercially available sulfane–sulfur probe (SSP4, Dojindo, Kumamoto, Japan) to detect endogenously generated polysulfides in biological samples [71,72]. Such sophisticated methods for the detection of H_2_S and now also for H_2_S_x_ in organisms may enable us to differentiate between the presence and the functions of H_2_S and H_2_S_x_– although -the design and development of such probes currently still poses a significant challenge in chemistry and in biology.

## 6. Conclusions

Polysulfides form a rather interesting class of small molecule redox modulators, in chemistry and biology. In some respects, these compounds are unique species found in vivo. First of all, polysulfides are entirely inorganic, which distinguishes them from the more common organic redox systems, such as quinones and flavonoids, and also from complexed metal ions and organic sulfur compounds. Secondly, they can be formed and transformed by multiple pathways, although not all of them may occur at the same frequency and under the same conditions. Thirdly, polysulfides may act as reductants and oxidants. And fourthly, they leave their mark on cells, as anti-oxidants and highly damaging (cyto-)toxic species, as inorganic signalling molecules, “cork-screws” for membranes, ligands for metal ions, inhibitors of proteins and enzymes and as a reservoir for inorganic sulfide (S^2−^). This facet rich behaviour of such small and simple molecules is truly astonishing and poses serious challenges for further investigations. The analytical fingerprints of polysulfides, for instance, are vague and unspecific, whilst the biological footprints are often hidden, reversible, perhaps going unnoticed and possibly also caused by other RSS, such as disulfides and H_2_S.

Despite these apparent obstacles, polysulfides remain fascinating molecules in their own rights. Their formation, occurrence, physical, and chemical properties, unique reactivities and biological impact are certainly worthy of our attention and consideration. Here, related species, such as HSSeSH and Se_n_S_8−n_, should also be accounted for, as there is now firm evidence for the formation and significant biological activity of such mixed “reactive sulfur selenium species”, together with the posttranslational modifications of selenocysteine residues, such as SeCysSeSH. Such studies are not purely “academic”, as sulfur represents a central player in human health, from its anti-oxidant potential to modulation of the microbiome, from nutrition to intra- and inter-personal H_2_S signalling. The scene is therefore set for some exciting discoveries in this field.

## Abbreviations

3-MST	3-mercaptopyruvate sulfurtransferase
CARS	cysteinyl-tRNA synthetases
CBS	cystathionine *β*-synthase
CSE	cystathionine *γ*-lyase
^∙^cPTIO	2-(4-carboxyphenyl)-4,4,5,5-tetramethylimidazoline-1-oxyl-3-oxide
CysSSH	cysteine persulfide
CysS_x_SH	cysteine polysulfides
DATS	diallyltrisulfide
DATTS	diallyltetrasulfide
DTPA	diethylenetriaminepentaacetic acid
GSH	reduced glutathione
GSSG	oxidized glutathione
GSSSG	glutathione trisulfide
GSSeSG	selenodiglutathione
HS_x_^−^/S_x_^2−^	inorganic polysulfides
H_2_SeS_x_	selenium disulfane
Nrf2	nuclear factor erythroid 2–related factor 2
NHE	normal hydrogen electrode
pDNA	plasmid DNA
Pr	protein
PrS-	protein thiol
PrSS-	protein persulfide
RSeS	reactive selenium species
ROS	reactive oxygen species
RSS	reactive sulfur species
RSSH	Hydropersulfides RS_x_R’
RS_x_H	organic polysulfanes
SOD	superoxide dismutase
UV-Vis	ultraviolet-visible
